# Experimental Correction and Treatment of Chronic Liver Failure Using Implantable Cell-Engineering Constructs of the Auxiliary Liver Based on a Bioactive Heterogeneous Biopolymer Hydrogel

**DOI:** 10.3390/gels9060456

**Published:** 2023-06-01

**Authors:** Murat Shagidulin, Nina Onishchenko, Victor Sevastianov, Mikhail Krasheninnikov, Aleksey Lyundup, Alla Nikolskaya, Alena Kryzhanovskaya, Sofia Voznesenskaia, Mariia Gorelova, Nadezhda Perova, Igor Kozlov, Artem Venediktov, Gennadii Piavchenko, Sergey Gautier

**Affiliations:** 1 Federal State Budgetary Institution “Shumakov National Medical Research Centre of Transplantology and Artificial Organs” of the Ministry of Health of the Russian Federation, 123182 Moscow, Russia; 2 Federal State Autonomous Educational Institution of Higher Education, “I.M. Sechenov First Moscow State Medical University” of the Ministry of Health of the Russian Federation (Sechenov University), 119435 Moscow, Russia; 3 Research and Education Resource Centre for Cellular Technologies, Peoples’ Friendship University of Russia (RUDN University), 117198 Moscow, Russia; 4 M.V. Lomonosov Moscow State Academy of Fine Chemical Technology (MITKhT), 119571 Moscow, Russia; 5 ANO “Institute Biomedical Research and Technology”, 123557 Moscow, Russia

**Keywords:** regenerative medicine, biomaterials, tissue engineering, gels, matrix, liver cells, multipotent mesenchymal stem cells from bone marrow (MMSC BM), cell-engineered constructs (CECs), chronic liver failure treatment

## Abstract

Our study sought approaches for chronic liver failure (CLF) treatment and correction via cell-engineered constructs (CECs). They are built from biopolymer-based, microstructured, and collagen-containing hydrogel (BMCG). We also strove to evaluate the functional activity of BMCG in liver regeneration. Materials and Methods: Allogeneic liver cells (namely, hepatocytes; LC) together with mesenchymal multipotent stem cells of bone marrow origin (MMSC BM; BMSCs) were adhered to our BMCG to compose implanted liver CECs. Thereafter, we investigated a model of CLF in rats receiving the implanted CECs. The CLF had been provoked by long-term exposure to carbon tetrachloride. The study comprised male Wistar rats (*n* = 120) randomized into 3 groups: Group 1 was a control group with the saline treatment of the hepatic parenchyma (*n* = 40); Group 2 received BMCG only (*n* = 40); and Group 3 was loaded with CECs implanted into the parenchyma of their livers (*n* = 40). August rats (*n* = 30) made up a donor population for LCs and MMSC BM to develop grafts for animals from Group 3. The study length was 90 days. Results: CECs were shown to affect both biochemical test values and morphological parameters in rats with CLF. Conclusion: We found BMCG-derived CECs to be operational and active, with regenerative potential. Group 3 showed significant evidence of forced liver regeneration that tended to persist until the end of the study (day 90). The phenomenon is reflected by biochemical signs of hepatic functional recovery by day 30 after grafting (compared to Groups 1 and 2), whereas structural features of liver repair (necrosis prevention, missing formation of vacuoles, degenerating LC number decrease, and delay of hepatic fibrotic transformation). Such implantation of BMCG-derived CECs with allogeneic LCs and MMSC BM might represent a proper option to correct and treat CLF, as well as to maintain affected liver function in patients with liver grafting needed.

## 1. Introduction

The issue of chronic liver failure (CLF) treatment is known to have no definitive answer yet. In particular, mortality remains high in the decompensation of liver diseases, with no considerable decline [1,2]. The total prevalence of irreversible liver pathology is now constantly increasing without decompensated liver failure. Currently, the only radical approach to solving this problem is liver transplantation. However, this advanced surgery is limited by a lack of donor organs, and the donor famine is predicted to be exacerbated [3]. The low efficiency of modern conservative care and surgical interventions is attributed to an early disability, especially in the young working population. At the same time, we should not ignore meaningful social and economic risks associated with the subject.

The modern state of the art defines chronic liver failure (CLF) as resulting from a critical decline in the number of viable and functionally active hepatocytes, which perish due to liver disease. Connective tissue growth interferes with hepatocytic proliferation. An excessive workload on remaining liver cells deteriorates their capacity to regenerate and divide, while connective tissue proceeds to occupy sparse zones for young hepatocytes so that apoptosis is provoked. Regenerative approaches have been recruited to stimulate the functional, proliferative, and anti-apoptotic abilities of cells in the organ with isolated allogeneic liver cells and allogeneic or autologic bone marrow mesenchymal stem cells. Their clinical effectiveness suffered, however, because of the rapid collapse of the cells. The need to develop grafted artificial organ analogues or cell-engineered tissue constructs (CEC) became apparent to replace impaired activities via cellular clusters inside them and to enable structural recovery of the damaged tissues. Such an artificial tissue requires both relevant cells and a matrix, which serves as a substrate to support the viability of adhered cells. Three-dimensional matrices are basic for the development of tissues; they improve proliferation and provide a constant shape and physical and mechanical properties for such a medical product. The integrative solution ought to develop and apply a clearly novel and efficient pathogenetic method to treat CLF [4,5,6,7,8,9,10,11]. Resulting cell-engineered constructs balance the lack of liver parenchyma cells, thereby leading to a longer period of validity. In either proliferation or apoptosis, the cells secrete numerous growth-stimulating factors to enhance the activity of the remaining cells. Finally, CLF signs are mitigated and corrected. The use of CEC guarantees donor cells’ survival, but the matrix, in particular, holds proper surrounding conditions.

Cellular technology and molecular biology have experienced eventful progress. As a result, we obtained unexpected opportunities to rebuild major lesions using artificial tissues obtained either in vitro or in vivo. Growth substrates should not only include cells but also three-dimensional matrices to provide cell adhesion and proliferation. They determine certain shapes of medical products as well as their physical and mechanical properties. Biological origin is preferable for these matrices to facilitate pointing, organization, synthetic and mitotic stimulation, and lineage selection when constructing a new operational tissue in bioartificial or tissue-engineered systems. Thus, impaired regions are settled by grafted cells, while the matrices act as scaffolds for substrate-dependent cells. In this way, they emulate the properties of the natural extracellular matrix and guide the course of tissue development [12,13]. Such biocompatible products should fulfill several obligatory requirements: absence of local inflammatory reactions to them; null toxicity, immunogenicity, carcinogenicity, or genotoxicity; the missing role of infection trigger; preservation of their properties during their entire shelf life.

Biodegradable matrices are principally defined by such regulated parameters as biodegradation time. The degradation should result in compounds with no biological hazards, maintain the growth rates of new operational tissues, and tend to be completely replaced by conventional tissues. Synthetic or natural hydrogels both seem to represent the most appropriate option among the implanted matrices. They imitate hydrated saccharidic components of the extracellular matrix. A critical advantage of these gels is their ability to closely wrap grafted cells to ensure a healthy microenvironment.

The state of the art in tissue engineering of the liver reveals the poor efficiency of modern conservative and surgical methods to treat CLF. Novel methods of high performance are in demand to improve the utility and prolong the regulatory impact of cell therapies using biocompatible and biodegradable matrices with donor cells. Biopolymer-based, microstructured, collagen-containing hydrogels (BMCG) respond to the mentioned requests. Their physicochemical, mechanical, and technological properties enable the use of gels to develop transitional frameworks for hybrid bioartificial organs [12]. For instance, hydrogels are multifunctional (acting at the same time as a scaffold, substrate, and growth medium for cell cultures), mechanically strong and elastic (that is convenient for surgeries), biocompatible at molecular and cellular levels, triggering proliferation and differentiation, porous (micropore size reaches 100–400 μm to enable new capillary growth), able to launch regeneration in neighboring tissues (in local administration), accessible to sterilization by usual methods without loss of medical and technical properties, and finally biodegradable (from 3–4 weeks to 6–9 months).

We have preferred a biopolymer-based, microstructured, and collagen-containing hydrogel (BMCG), which is bioidentical to body tissues and then biocompatible [14,15,16,17,18].

Our study was intended to reveal if it is a proper choice to correct and treat CLF via BMCG-derived cell-engineered constructs (CECs) and to evaluate BMCG’s functional activity to permit liver regeneration.

## 2. Results and Discussion

**Figure 1:**
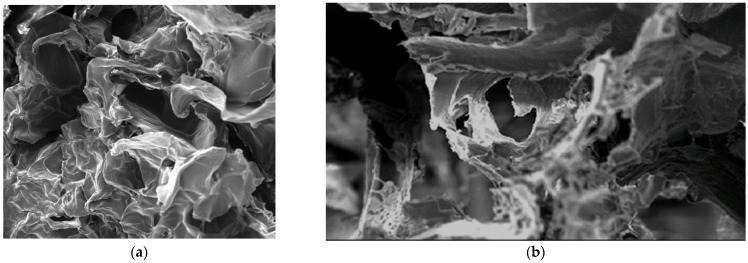

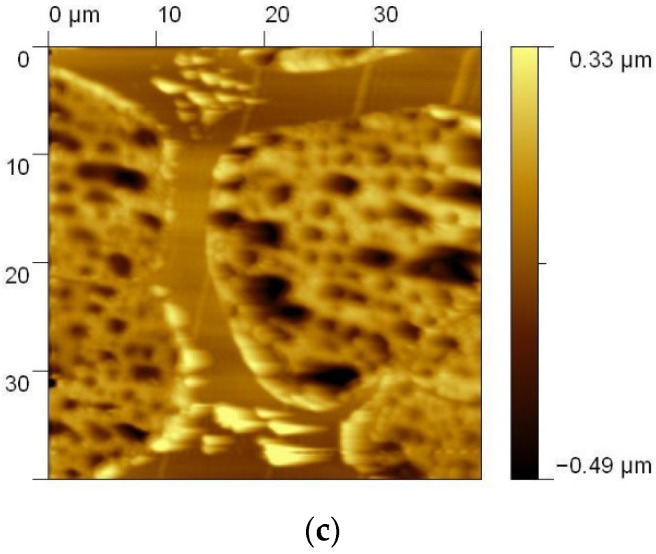
Microstructure of the BMCG surface. Scanning electron microscopy. JSM-6360 LA (Jeol, Tokyo, Japan): (**a**)— magnification ×100; (**b**)—magnification ×300; (**c**)—an image of the BMCG surface after cutting at −80 °C by scanning probe nanotomography. The image size is 40 × 40 μm.

We examined the external structure of our BMCG by optical microscopy and SEM. Here, the BMCG is condensed in microparticles with their sizes ranging from ~35 to 300 μm on average (we only used those of 145.79 ± 0.09 μm) suspended in collagen solution with a complex relief of the gel surface (Figure 1). Samples of the bioactive heterogeneous biopolymer-based hydrogel were investigated in situ immediately after ultrathin cutting by a cryo-ultramicrotome; we detected hydrogel particles with a porous microstructure (Figure 1b).

**Figure 2:**
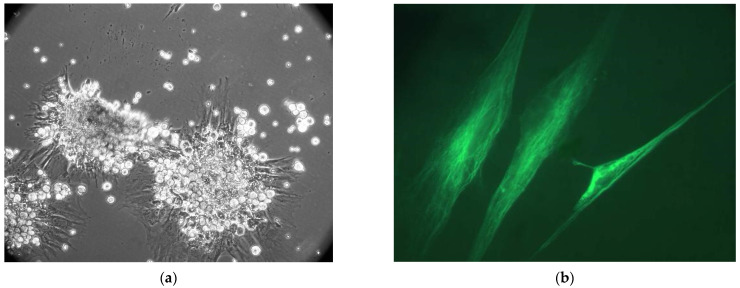

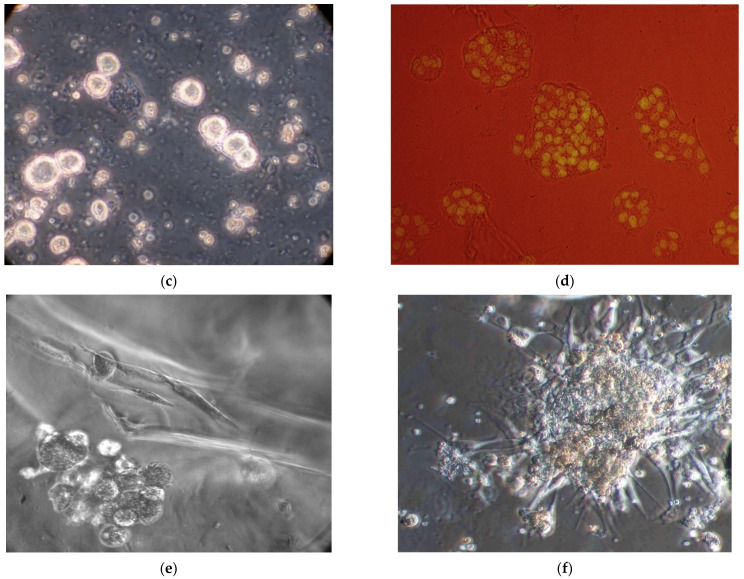
(**a**)—MMSC BM culture. The cultivation period was 7 days. Phase-contrast microscopy. Magnification ×100; (**b**)—MMSC BM stained by anti-vimentin antibody (J144), IF, Magnification ×630; (**c**)—obtained hepatocytes, phase contrast, Magnification ×400; (**d**)—hepatocytes, fluorescence staining + phase contrast by hepatocyte nuclear antigen 4α (HNF-4α), Magnification ×200; (**e**)—cocultivation of LCs and MMSC BM for 10 days Magnification ×400; (**f**)—active LC attachment (spreading) onto a single-layered surface of MMSC BM. Nomarski microscopy. Magnification ×200.

We carried out a comparative efficiency evaluation for implanted CECs with BMCG, allogeneic LCs, and MMSC BM (5:1) in CLF treatment. The timing of survival as well as dynamics were assessed in the recipient’s livers by day 90; we also explored the biochemical profile and morphological state. The survivability of MMSC BM inside our primary cell culture reached 94%, with a standard error of 2%. Suspended cells obtained from donor rodent livers contained ~95–98% of hepatocytes and ~2–5% of stromal cells, with a survival rate of 76 ± 4%. We did not perform cell segregation into parenchymal and stromal pools. The primary cell cultures underwent precultivation to avoid stress damage to isolated cells and to boost their activity. BMSCs experienced 7-day-long cultivation followed by a cocultivation with LCs added for three subsequent days (Figure 2). We realized cocultivation in both static and dynamic modes (1.5–2.0 rotations/min) to simulate the phenomena of a strong transport flow that is typical in blood circulation. The conditions exclusively provided long-term LC survival with no loss of mitotic activity in cocultivation with MMSC BM (Figure 2f).

**Figure 3:**
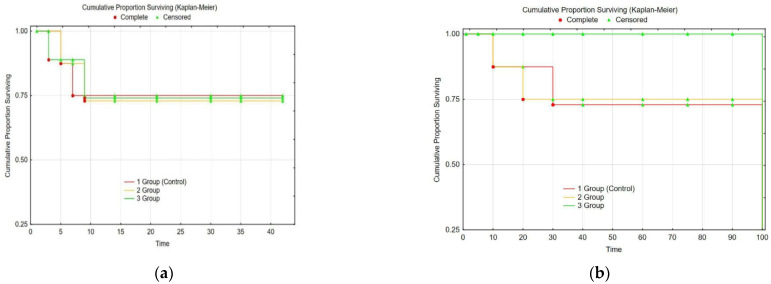
Animal survival in the study (Kaplan–Meier): (**a**)—CLF modeling; (**b**)—CLF treatment by BMCG-derived CECs.

The CLF model led to 25% of fatal outcomes in 42 days. Groups 1 and 2 each included 5 rats that died during the whole study period of 90 days (from 40 animals in every group). We estimate this to prove the choice of the CLF model’s severity to be proper. Group 3 exhibited no lethal cases for the same period (42 + 90 days). The rat’s survival after poisoning and treatment application is illustrated in Figure 3.

**Figure 4:**
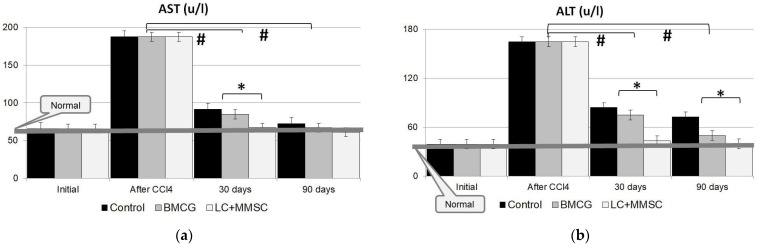

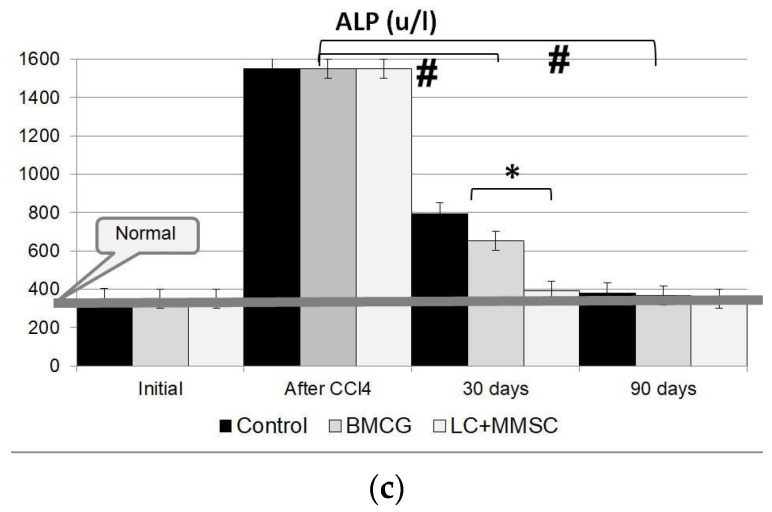
Dynamics of hepatic enzyme levels in blood sera of the rats: (**a**)—AST, (**b**)—ALT; (**c**)—ALP in CLF modeling with CEC implantation. Group 1—control (CLF + 1 mL saline infusion); Group 2 —experimental group (CLF + 1 mL of BMCG); Group 3—another experimental group (CLF + 1 mL of BMCG-derived CECs containing allogeneic LCs and MMSC BM as 5:1 + Cytodex-3). *—the difference is considered significant compared to the enzyme levels of Groups 1 and 2; *p* < 0.05; #—the difference is considered significant compared to original enzyme levels after CLF modeling; *p* < 0.05.

In animals with CLF, we assessed a reversion of functional recovery in the liver by a biochemical recovery in the peripheral blood: ALT, AST, and ALP. Those markers tended to increase sharply immediately after the end of poisoning for all the groups (Figure 4a–c). By day 7 after the last poison intake, AST content had increased by 4.5 times or more, while that for ALT had only increased by 3 times or more; finally, ALP had been elevated by almost five times. Group 3 (with implanted CECs) maintained a higher rate of recovery than Groups 1 and 2. Specifically, the values of ALT, AST, and ALP in Group 3 returned to their initial meaning entirely in the first 28–30 days, with no further change until the end of the study (Figure 4). Group 1 had significantly elevated thresholds for the values for 90 days; moreover, additional mortality remained remarkable at this time (Figure 2 and Figure 3). The poisoning is completed; in seven days, AST increased by 4.5 times or more while ALT was more than three times elevated, with alkaline phosphatase increasing by hardly five times.

The study showed that biochemical recovery (ALT, AST, and ALP) occurred in Group 3 during the first 28–30 days with no pathological changes, whereas the control group (Group 1) and Group 2 retained elevated levels of the markers for 90 days (Figure 3). An extra number of fatal outcomes also occurred in Groups 1 and 2 (Figure 2b).

**Figure 5:**
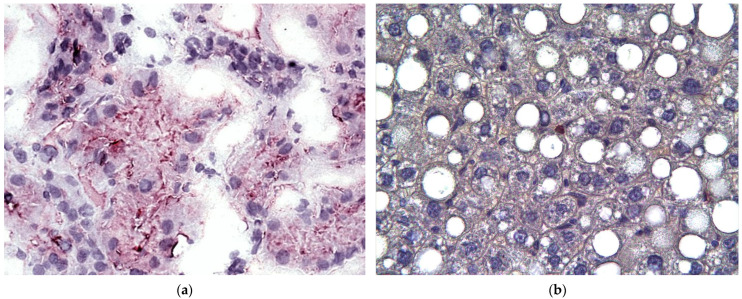

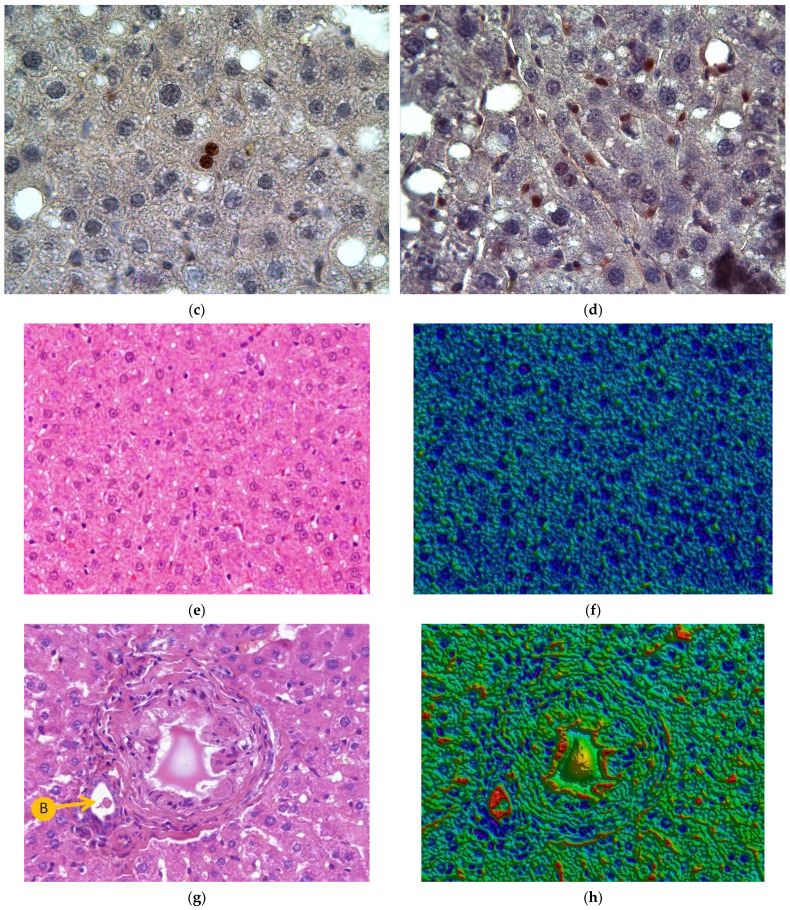
(**a**)—viable hepatocytes inside CECs. Immunohistochemical study by cytokeratin-18 antibodies (CK-18): slight positive focal staining with poor intensity in the cytoplasm. Magnification ×400. (**b**)—PCNA staining for Group 1 with no treatment: a negative reaction. Magnification ×400. (**c**)—PCNA staining for Group 2 with CECs only: local positive staining. Magnification ×400. (**d**)—PCNA staining for Group 3 with grafted LCs and MMSC BM with BMCG-derived CECs: a pronounced positive reaction, numerous viable hepatocytes. Magnification ×400. (**e**–**h**)—rat liver by Day 90, Group 3 after last poison intake for CLF modeling: (**e**)—structural hepatic recovery, architectonics is almost similar to a normal liver morphology. No dystrophic signs in hepatocytes. The localization of plates is fairly physiological. Hematoxylin-eosin stain, Magnification ×200; (**f**)—artificial pseudostaining of image (**e**), blue color indicates the cell nuclei, dark green—cytoplasm, light green—spaces; (**g**)—rat liver at CEC grafting zones (Group 3, LCs + MMSC BM = 5:1) by Day 90. Bile duct recovery (B) at the periphery of CECs. Hematoxylin-eosin stain, Magnification ×200; (**h**)—artificial pseudostaining of image (**g**), blue color indicates the cell nuclei, green—cytoplasm; red—empty spaces Magnification ×200.

Our histological assessment of toxically damaged livers (Figure 5) with CLF modeling exhibited a necrotic shift in the LC structure with fatty atrophy. The described phenomenon manifested itself in a large number of hepatocytes. Nuclear degeneration accompanied the process, as did intranuclear lipid inclusions. It is interesting that the number of binucleated hepatocytes was found to be significantly reduced. In addition, we detected glycogen disappearing from the hepatocytic cytoplasm. Parenchymal cells exhibited pronounced polymorphism. Some hemorrhagic foci in the parenchyma were present. Additionally, adipose and vacuolar dystrophy appeared at many sites in the liver cells. In addition, we can pay attention to connective tissue gains associated with liver fibrosis development (namely, periportal and portocentral fibrotic changes) together with considerable lymphocytic infiltration of the same areas. Some false lobules were also notable (Figure 5).

On day 90 after CLF modeling, we observed a subtotal reorganization of tissue architectonics with a shift from normal parenchyma to false lobules in the rat livers of our control group. Sclerotic changes (fibrosis) manifested themselves by emergent collagen fibers along the portal tracts as well as by portoportal and portocentral septa simultaneously with the formation of false lobules. A pronounced protein-associated hepatic dystrophy was present. We also noted focal necrosis in hepatocytes and sclerotic and fibrotic changes in the parenchyma. In addition, there were plenty of sinusoids and central veins. In addition, rare lymphocytic infiltration accompanied by young proliferating histiocytes was observed while fibrosis progressed.

We used Cytodex-3, which had been added to our bioactive heterogeneous biopolymer-based hydrogel (BHBH), to prove that its matrix is not beyond biodegradation and to highlight the grafting zones of CECs in the liver. We found BHBH to almost completely disappear by day 90 on slides with confirmed biodegradability. Group 2 revealed the same structural changes as Group 1. Except for this, connective tissue arose in the presence of Cytodex-3. We might explain this fact as a tissue reaction to foreign bodies such as Cytodex-3.

Group 3 displayed some angiogenesis and bile ductule recovery on day 90 after CEC grafting into the liver (LCs + MMSC BM) (Figure 5g). The grafted hepatocytes at the CECs were viable. Positive cytokeratin-18 staining verified this phenomenon.

By this time, allogeneic donor cells, which had been previously cultivated together with MMSC BM and then grafted via CECs (cell clusters among the BHBH matrix), had not only maintained their viability but also functional activity at the grafted CECs. Moreover, they integrated to constitute recipient hepatic tissue with no considerable inflammatory reaction or rejection signs.

The liver morphology assessment in Group 3 revealed a pronounced positive shift toward recovery on day 90. Histological liver studies for this group showed CEC-free areas to be completely repaired (Figure 5), with no evident difference from a physiological state. We observed fatty degeneration to regress in hepatocytes and to obtain identity with fine droplets, which is generally considered more favorable and reversible. Furthermore, intact hepatocytes appeared with no signs of perivenous dystrophy.

Overwhelming results were seen with PCNA immunohistochemical staining of liver slides for all three study groups. Marking nuclear signs of new proliferation, the dye was almost completely degraded in the no treatment state, with an already demonstrated sorrowful image for Group 1: active fibrotic development, abundant fatty dystrophy, and no relevant liver plates in the lobular structure. For Group 2, PCNA processing revealed some positive shifts, such as diminishing replacement by adipose tissue (despite the well-mentioned ubiquitous lipid droplets in the cytoplasm) without any recovery of fibrosis but with solitary hepatocytes and even binucleated hepatocytes. Finally, we discovered signs of an intense “second wind” for Group 3 in the PCNA treatment of the slides: the general pattern tended to include dozens of developing cells alongside the sinusoids so that recovered plates became remarkable. Interestingly, in this case (PCNA IHC), the final checkpoint was not defined by a full recovery with some retained signs of dystrophy; however, the trend remained evident.

**Figure 6:**
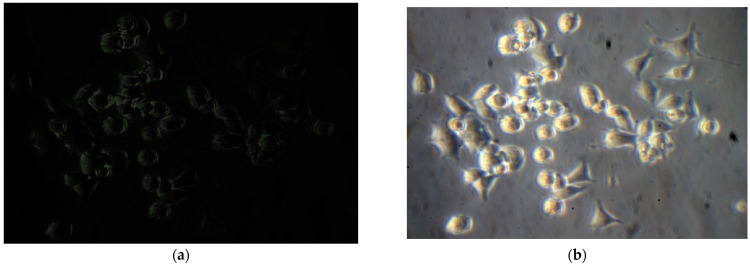
PC-12 cell line after 3 days of cultivation without no nerve growth factor. No fluorescence in anti-vimentin staining. Magnification ×400. (**a**); phase contrast. Magnification ×400. (**b**).

In the present study, the following negative controls were carried out for our ICC/IHC staining. First, an anti-vimentin control was provided with murine pheochromocytoma cells (Figure 6).

**Figure 7:**
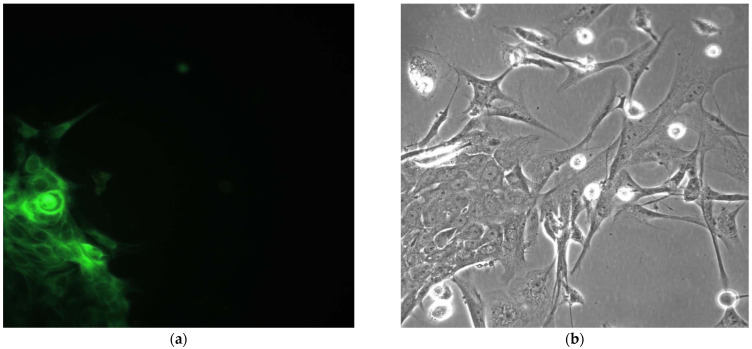
Co-cultivated MSC and pancreatic beta-cells. Anti-HNF-4α fluorescent. Magnification ×630 (**a**) and phase contrast reactions. Magnification ×630 (**b**). Positively stained epithelial cells in ICC staining.

For an anti-HNF-4α negative control, we cocultivated MSC and pancreatic beta-cells (Figure 7). Mesenchymal origin gives no positive staining, while epithelial cells reveal an evident reaction.

**Figure 8:**
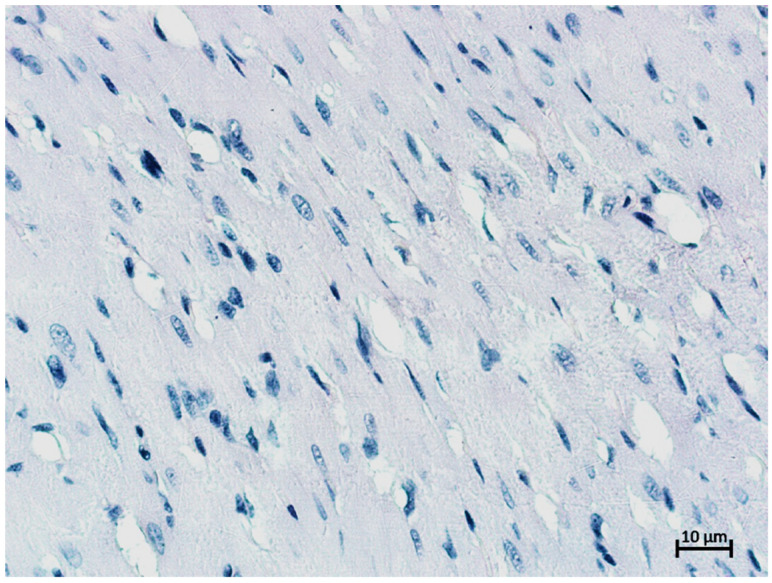
Cardiac muscle cells of a rat. Anti-CK-18 IHC reaction. Additional haematoxylin staining. Magnification ×200. No positive reaction.

Cytokeratin-18 (CK-18) is a type I intermediate filament protein responsible for maintaining a cellular structure that is expressed in epithelial cells, including sinusoidal endothelial cells [19]. CK-18 is a major protein of liver intermediate filaments, and CK-18 aggregates are major components of Mallory bodies; embryonic hepatocytes contain cytokeratins 8, 18, and 19, but mature cells only contain CK8 + 18, while CK-19 becomes negative by the tenth week of pregnancy [20,21]. Stromal and vascular endothelial cells were found to have immunonegative staining, resulting in a total percentage of tissue unresponsive to antibodies for CK8 + 18. Cardiac muscle tissue was used as a negative control (Figure 8).

**Figure 9:**
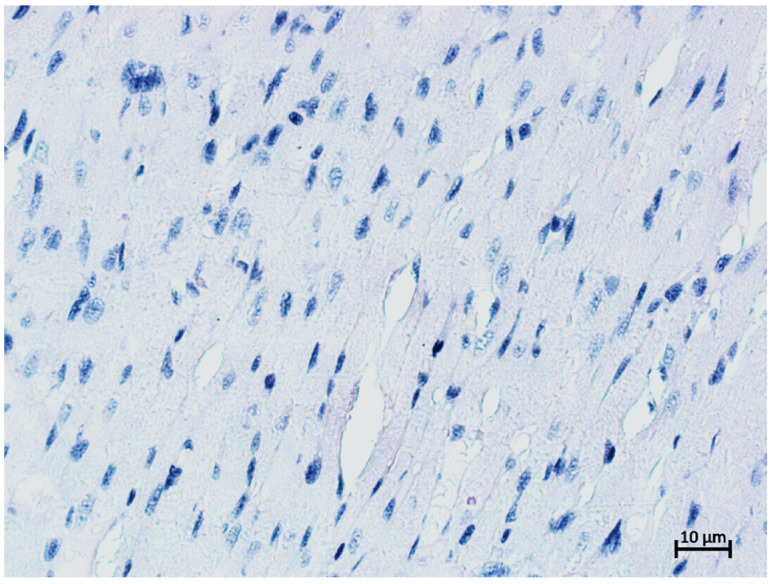
Cardiac muscle cells of a rat. Anti-PCNA IHC reaction. Additional haematoxylin staining. Magnification ×200. No positive reaction.

For the PCNA negative control, we also chose cardiac muscle, as adult cardiac muscle cells are known to be non-regenerating (Figure 9).

**Figure 10:**
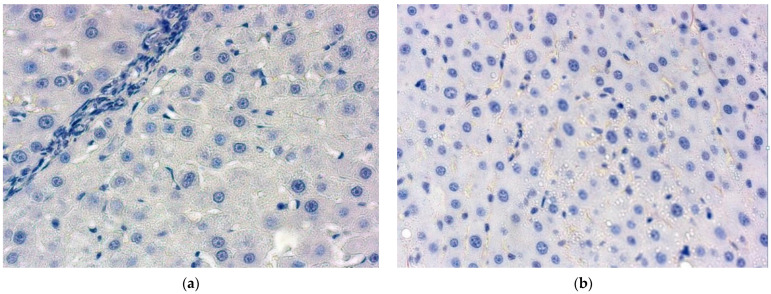
Liver sections from Group 3 rats (an almost entire liver recovery in BMCG with allogeneic LCs and MMSC BM 5:1). Isotype controls for the anti-CK-18 MCA + haematoxylin staining. Magnification ×400 (**a**); anti-PCNA MCA + haematoxylin staining. Magnification ×400. (**b**). No positive reaction in both cases.

We also provided the following isotype controls for the IHC staining to check if antigen-antibody binding for the experimental rats was really specific compared to MCAs of the same Ig subtype and conjugation manner for the same species (Figure 10).

**Figure 11:**
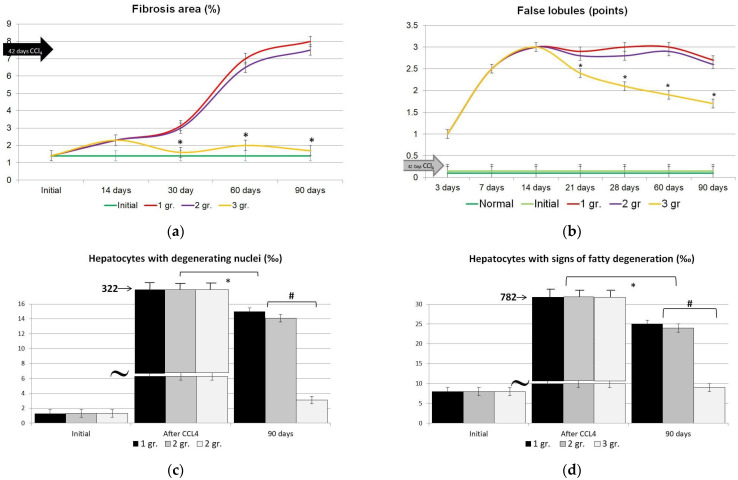

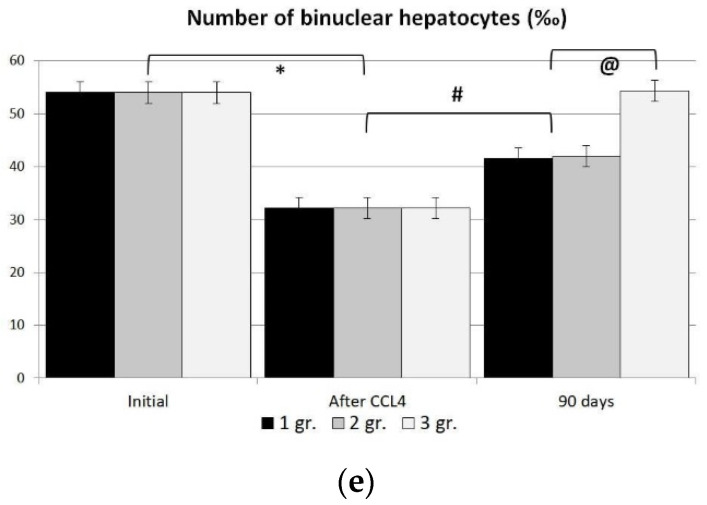
Dynamic morphometric assessment of nonparenchymal structures in CLF modeling without and with grafted CECs by day 90. (**a**)—changes in specific areas of connective tissue (%). In intact animals, the average size of the area reached 1.4%. (**b**)—changes in the number of false lobules in the liver. * The difference is significant compared to the values in control rats (Group 1); *p* < 0.05. (**c**–**e**)—dynamic morphometric assessment of rat hepatocytic parameters in CLF modeling without and with grafted CECs by day 90: (**c**)—hepatocytes with degenerating nuclei; (**d**)—hepatocytes with fatty degeneration; (**e**)—binucleated hepatocytes; Group 1—control (saline infusion); Group 2—BMCG matrices, Group 3—CECs with allogeneic LCs & MMSCs 5:1. *— the difference is considered significant comparing to the values in rodents from the control group (Group 1); *p* < 0.05. #—the difference is considered significant compared to the values in rats after CCl_4_ intake, *p* < 0.05; @—The difference is significant compared to the values in rats Group 1 and 2; *p* < 0.05.

We performed a quantitative and semiquantitative assessment of structural changes in CLF either without treatment or at CECs to reinforce our results. We studied liver parenchyma cells (counting the number of hepatocytes with fatty degeneration, degenerating nuclei, and intranuclear lipid inclusions and checking the number of binucleated hepatocytes). We evaluated nonparenchymal structures as well (connective tissue area size and number of false lobules). The values were recorded as relevant for day 90 after the end of CLF modeling (Figure 11).

A morphometric study of nonparenchymal structures (connective tissue area size and number of false lobules) over a 90-day period showed that the liver parenchyma recovered in the presence of CECs; likewise, the connective tissue area size was reduced (Figure 11a), as was the number of false lobules (Figure 11b). Simultaneously, the control group had an elevation of 8.2% and 2.8%, respectively.

We also found that CEC treatment (experimental Group 3) resulted in a rapid and considerable decrease in LC number with signs of fatty degeneration, degenerating nuclei, and intranuclear lipid inclusions. An increase in binucleated cells was higher. The modifications had a pronounced clinical benefit.

An analysis of the results indicated that immediately after the last poison intake, the levels of cytolytic enzymes sharply increased in animals in all three groups. However, Group 3 with CEC implantation had a higher rate of recovery than the control group (Group 1) and Group 2. The values of ALT, AST, and ALP for Group 3 reached their initial values during the first 28–30 days and remained at this level until the end of the study (Figure 4). Group 1 had a considerable elevation of the parameters for 90 days of our observation, and fatal outcomes were fairly frequent at this time (Figure 2 and Figure 3). Thus, CECs with LCs and MMSC BM led to a faster decrease in ALT, AST, and ALP levels in the blood sera of rats with CLF than the approaches for Groups 1 and 2.

Allogeneic donor liver cells (cocultured with MMSC BM and grafted as CECs with a bioactive matrix of BHBH) not only retained their viability (Figure 5a) and functional activity (Figure 4) but integrated into the hepatic tissues in recipients with neither pronounced inflammatory reactions nor rejection signs. The high rate of hepatic marker recovery during the first 30 days in CLF and CECs indicated that a long-term dynamic study was required not only for liver tissues but also for CECs. This would identify the potential of CECs to be continuous centers of induction and maintenance of regeneration in liver disease.

We performed a comparative study of morphological recovery in the liver after CLF modeling in CEC-intact zones. Surprisingly, by day 30, Group 3 had a considerably lower incidence of toxic liver damage than Groups 1 and 2. The phenomenon is manifested by the regression of fatty dystrophy with a shift to fine droplets in cells (a phenotype that is more favorable and reversible). We also observed a recovery of lobular structure with intact hepatocytes around the veins and a reconstruction of plate architectonics. The plates in CLF modeling and CEC-grafting recovered by day 30 with no evident difference from a normal state. False lobules were not notable, whereas Groups 1 and 2 still had necrotic foci with some false lobules (Figure 5b–d, especially).

By day 90 of CLF modeling in Group 1, a subtotal reconstruction of hepatic tissue architectonics with false liver lobules had occurred. Sclerotic shifts are manifested by the growth of collagen bundles alongside the portal transport system and by portoportal and portocentral septa together with false liver lobules. Focal necrosis of hepatocytes was present. Sinusoids were expanded and extensively filled by blood as well as central veins. A few lymphocytic infiltration sites were noted in the parenchyma. Young and mature histiocytes proliferated there with no new bile duct.

A comparative assessment of liver morphology on day 90 of CLF modeling and CEC implantation (Group 3) showed pronounced positive dynamics of hepatic parenchyma recovery (Figure 5e). A histological study in this group emphasized that the liver architectonics had completely recovered by this time in CEC-intact areas compared to the control, with almost no difference from a normal liver. Hepatocytes had no signs of dystrophy, whereas our control group exhibited a pronounced protein-associated dystrophy of hepatocytes with sclerosis and fibrosis in the parenchyma. Histological description of the implanted CECs and surrounding liver tissues on day 90 after grafting indicated the presence of viable hepatocytes. The cells proliferated intensely, while new blood vessels grew and recovered bile ductules were found (Figure 5g).

A faster rate of structural recovery in damaged liver tissue, a greater extent of connective tissue area size regression, and a decrease in cell number of those with fatty degeneration and destroyed nuclei on day 90 in Group 3 proved, in our opinion, that CECs (with LCs and MMSC BM at a ratio of 5:1) were able to affect regenerative processes in liver tissues. This might be due to a more effective morphofunctional reconstruction of CECs after their implantation. The results indicate that grafted LCs of CECs (in cases where LC + MMSC BM = 5:1) retain their viability and proliferative activity for a long time (at least 90 days). The CECs integrate into a recipient’s liver, and this occurs without any pronounced inflammatory reaction or signs of rejection, even if there is no immunosuppression.

The aforementioned points lead us to recognize that grafted CECs may serve to constitute *de novo* centers of reparative liver regeneration. The centers seem to be able to ensure their prolonged functioning and thereby provide bioregulation of tissues in the damaged liver. Thus, the obtained results of the biochemical, histological, morphological, and morphometric studies in our CLF model demonstrate the functional activity of BHBH-derived CECs. Our analysis of the results unwraps our technology for further use in clinical studies in patients with extremely severe forms of CLF.

## 3. Conclusions

The rat model of CLF revealed a liver-located implantation of experimental CEC samples with LC/MMSC 5:1 and BMCG-matrix to help liver recovery for a long time. The phenomenon includes a rapid recovery of cytolytic liver enzyme content in the blood (by Day 30) and a recovery of tissue phenotype (a necrosis eradication, a reduction in the number of damaged hepatocytes, and a decrease in connective tissue area). Grafted CEC proved to maintain a functional balance of cells (LC and MMSC symbiotically) in the liver for all of the study time. The results of the studies imply that CEC-associated LC and MMSC can transform the constructs into permanent hepatoid tissue development centers via the production of bioregulatory factors with autostimulation and parenchyma stimulation. We have finally come to an understanding of BMCG-derived CEC with allogeneic LC and MMSC as an efficient CLF-modifying method. The technology also serves to support hepatic functions in those who attend the organ’s transplantation.

The main limitation of the present study is its current difficulties with translation to clinical practice. Russian legal practice avoids permitting the use of allogeneic cells, so the approach is locally limited for clinical implementation.

Cytodex-3 is not likely to be used in the technology clinically, as it has already been hired as a CEC injection site marker in the late period (90 days) of our study.

## 4. Materials and Methods

### 4.1. Heterogeneous Hydrogel: Description and Preparation

We used a biopolymer microstructured collagen-containing hydrogel (BMCG) as a cell carrier to build cell-engineered constructs in the liver (it was a commercially available injectable heterogeneous BMCG with a complex of peptides and glycosaminoglycans) [14,15] (trademark Sphero^®^*GEL*, TU 9398-001-54969743-2008, FSR 2012/13033 dated 15.07.2015, manufacturer AO “BIOMIR servis”, Krasnoznamensk, Russia). This gel acts as a biomimetic of the extracellular matrix (ECM). Its homogeneous phase comprises normal ECM components (partially hydrolyzed collagen, proteoglycans, and glycoproteins), as well as a number of other bioactive substances, including peptides, amino acids, uronic acids, monosaccharides, etc. The heterogeneous phase consists of microparticles from cross-linked type I collagen with an average size of ~150 µm. The ability of BMCG to induce cell adhesion, proliferation, and differentiation has been previously shown in vitro and in vivo [16]. That is why we decided to use it as a bioactive matrix for the creation of cellular and tissue-engineered constructs [17,18]. A sample of the BMCG is a ready-to-use solution for injection (volume of 1.0 mL) in a syringe inside a double surgical sterile package (sterilized by radiation). BMGCs are currently approved for medical use.

### 4.2. Animals, CLF Model

Experimental studies included male Wistar rats (*n* = 120) aged 6–8 months with a body mass of 230–250 g and male August rats (*n* = 30) aged 5–6 months with a body mass of 150–230 g. The animals were held in our vivarium with a constant room temperature of 18 to 20 °C while a mixed diet and free access to water were provided. The animal studies were being conducted between 9:00 and 19:00 at 22 to 24 °C; thus, the periods included time-dependent fluctuations of cellular proliferative activity. All manipulations with rodents were implemented and carried out according to the rules recommended by the European Convention for the Protection of Vertebrate Animals used for research and other scientific purposes (European Convention for the Protection of Vertebrate Animals Used for Experimental and other Scientific Purposes (ETS 123), Strasbourg, 1986).

We achieved the CLF-model via prolonged poisoning of the healthy rats of the Wistar breed (*n* = 120) with CCl4, as it had been shown in our own scheme for intake periods of 42 days [13].

#### Euthanasia

Euthanasia was performed on days 1, 3, 7, 14, 21, 28, 60, and 90 by intraperitoneal administration of sodium thiopental at doses that caused respiratory arrest. By the Institutional Review Board (Ethics Committee) of the Federal State Budgetary Institution “Shumakov National Medical Research Centre of Transplantology and Artificial Organs” of the Ministry of Health of the Russian Federation (protocol code 050221-1/4e 05/02/2021). Immediately after euthanasia, we collected the animal blood; the livers were removed with a macroscopic study to take some pieces into 10% buffered formalin.

### 4.3. Obtaining LC and MMSC BM

We employed 5–6-month-old male rats of the August breed (*n* = 30) with a body mass of 150–230 g as donors of allogeneic LCs and MMSC BM for Group 3. Cell isolation and cultivation were kept according to the common principles of cultural studies. We prepared our cultures of MMSCs from the bone marrow via the generally distributed method [22,23]. Our culture of LCs was achieved thanks to the known procedure [22].

Initially, we established a starting cocultivation of isolated liver cells (2.5 to 4.0 × 10^6^ cells/cm^3^) and bone marrow MMSC (0.5 to 0.8 × 10^6^ cells/cm^3^) for three days on William’s E growth medium (REF 22551-022) at a ratio of 5:1 (LCs + MMSC BM) [13]. An additional 150 µL volume of BMCG suspension was added to the culture of cells to improve adhesion. We used 150 µL of Cytodex-3 (Sigma-Aldrich company Product No: C3275, No CAS: 88895-19-6, MDL No: MFCD00130902) to warrant the viability of grafted cells inside CECs for a longer time after the grafting. We adjusted the final volume of the suspension with LCs, bone marrow MMSCs, BMCG, and, also, Cytodex-3, up to exactly 1 mL by our William’s E growth medium (REF 22551-022). Then, this product was given to rats by injection into their livers. The ability of isolated, conserved, and cocultivated allogeneic liver and bone marrow mesenchymal stem cells to survive together was checked before implantation and verified via staining with trypan blue [24,25].

### 4.4. Group Description and Study Design

By day 7 since the last CCl_4_ intake, we had randomized the surviving rats (*n* = 120) into three groups. Thereafter, the CLF correction was checked for its efficiency in CEC grafting into impaired hepatic tissues: Group 1 stayed for a control group (CLF + 1 mL of saline, *n* = 40); and Group 2 was an experimental group (*n* = 40) (CLF + 1 mL of BMCG, *n* = 40); Group 3 was another experimental group (*n* = 40) where CECs (BMCG-derived and involving allogeneic LCs and MMSC BM at 5:1 with Cytodex-3 added) were to enter the liver (after stirring into a volume of 1 mL) by day 49 after the start of CLF modeling. The ratio between LCs and MMSC BM (5:1) was preferred due to its best results in our previous studies [13].

We verified whether the models had been relevant (as well as the efficiency of CEC-dependent structural swaps in the liver). For this, we inspected the rats’ mortality, survival, and hepatic morphological and morphometric features. Biochemical data for peripheral blood destined to test the functional effects of CECs. In CLF (either with or without treatment), animals were killed on Day 90 intraperitoneally by sodium thiopental solution at doses that caused respiration arrest. The livers were removed on days 1, 3, 7, 14, 21, 28, 60, or 90 after the last CCL_4_ intake. We investigated rat blood samples during the same observation period. Finally, morphological studies of liver sections were provided. We did not induce any immunosuppression during the experiments.

### 4.5. Parameters to Study

Our BMCG matrix has been studied by low-temperature scanning probe nanotomography after getting an ultrathin slide cut at an ultramicrotome at −80 °C. We employed a Leica EM UC6 microtome with a Leica EM FC6 camera to obtain ultrathin (20–100 nm) sections of BMCG-samples (the ultramicrotome has also been used in low temperatures). Liquid nitrogen was used to frost. We performed the sections with a special diamond DIATOME cryoAFM 35 knife with a 2 mm thick blade. In situ surface measures of the BMCG sample (immediately after the ultrathin sections) were recorded by an experimental prototype of low-temperature scanning probe nanotomography (SPN) inside the ultramicrotomic cryocamera. A magnet holder fixed the matrix gel to the SPN device. Then, we placed a silica resonator with SPN, which rendered nanoscale-visible surface topography. An SPN EG-3000M device for numerical control to take measures and process the results was used together with NSpec 7.0 software of Nano Scan Technology (Dolgoprudny, Russia); the sections and measures were recorded at −80 °C.

We used MATLAB software to enable three-dimensional surface imaging from histological photographs. Several structural patterns were identified: “oxyphilic liver cell cytoplasm”, “basophilic liver cell nuclei”, “fibrous connective tissue”, “intercellular media”, and “empty zones (vascular lumina or fat cells)”. Then, the original images were converted into grayscale with further three-dimensional surface building with respect to added constants for every grayscale image. The choice of models and colors was specific to each of the images [26]. Rats got anesthetics (ether) to obtain some peripheral blood from their tail veins for biochemical study via the tail tip notch. We examined liver function (e.g., ALT, AST, ALP, total protein content, and total bilirubin) by Beckman Coulter, Inc. biochemical analyzer AU 680 (USA) (REF: AST-OSR 6109, ALT-OSR 6107, ALP-OSR 6104, albumin-OSR 6102, bilirubin-OSR 6112). Fluorescent staining of anti-human HNF-4α (Life Technology, Carlsbad, CA, USA) with detection of goat anti-rabbit IG, Alexa Fluor (lot 74002428A, ref. 417700). Used anti-Vimentin antibody (J144; Santa Cruz Biotechnology, lot H2802, ref. 53464).

### 4.6. Liver Histopathological and Morphometric Analysis

We fixed the liver tissue samples in 10% buffered formaldehyde, further embedding them in paraffin. Histological studies took place on days 1, 3, 7, 14, 21, 28, 60, and 90. Thin paraffin sections (5 µm) were cut and then stained with routine hematoxylin and eosin staining (HES). A Leica microscope of the DM 6000 B model (Wetzlar, Germany) and a Leica camera of the LTDCH 9435 model (same producer) were used.

IHC reactions were provided with MCA to proliferating cell nucleus markers, mouse anti-PCNA (PC10; Invitrogen, lot 1534324A, ref. 180110), and cytokeratin 18, CK-18 (Cytokeratin 8/18 Antibody 5D3; Invitrogen, lot RL2314941B, ref. 11344983). Deparaffinized tissue sections were then pretreated via citrate buffer for 10 min. Then, we incubated these sections with MCA for 30 min at room temperature. We employed the labeled streptavidin-biotin method (DakoCytomation, Hamburg, Germany; LSAB) with a detection system (biotin-streptavidin complex and peroxidase) to reveal if there was an immune interaction (a set of 10-min-long incubations in the presence of biotinylated link antibody together with peroxidase-labeled streptavidin). Meanwhile, diaminobenzidine served as a visualization substrate. Finally, our sections got an additional HES. All the controls required were obtained, too.

The morphometric study of liver cells exploited HES sections. Camera-equipped light microscopy was continued by software analysis via ImageScopeM (“Systems for microscopy and analysis”, Russia; LeicaDM1000 and Leica LTDCH9435 microscopes; DFC 295 camera by Leica Camera AG, Wetzlar, Germany). The micrographs were taken with a magnification of ×400 via the ImageJ software package [27,28]. We measured the total exposed area of hepatocytes with nuclear areas to calculate the areas of their cytoplasm. We registered the numbers of mono- and binucleated cells, counting for their relative presence normalized to a single unit of area measurement.

### 4.7. Statistical Analysis

We processed the results with the Biostat software; we used a *t*-test to verify if the differences remained significant with respect to Bonferroni correction. The differences were found to be significant at *p* < 0.05 (the package is recommended by WHO, EpiInfo 5.0).

The animal rate of survival was studied by the Kaplan and Meier curve via the “Statistica software for Windows v. 12” statistical package. Comparisons of the survival rates of the groups were evaluated by the log-rank test, where *p* values less than 0.05 were considered to be significant.

Our final statistical result processing was performed in the “R software” computing environment. The distribution models were defined by the Shapiro-Wilk test. We calculated confidence for the studied parameters to note differences between two compared groups by Wilcoxon and t tests with respect to the Holm-Bonferroni correction.

## Data Availability

The data presented in this study are available on request from the corresponding author.
